# Analysis of prescription status of antihypertensive drugs in Chinese patients with hypertension based on real-world study

**DOI:** 10.1080/07853890.2022.2162113

**Published:** 2023-01-03

**Authors:** Renren Yang, Jia Tang, Ming Kuang, Hongying Liu

**Affiliations:** Medical Affairs, Hangzhou Kang Sheng Health Consulting CO., Ltd, Hangzhou, China

**Keywords:** Antihypertensive drugs, prescription, hypertension, treatment pattern, real-world study

## Abstract

**Background:**

Globally, the prevalence of hypertension and the accompanying burden of cardiovascular disease are increasing. Through drug utilization research, we can understand the prescription status of hypertension patients and promote rational drug use. The purpose of this retrospective study was to describe the current prescription pattern of antihypertensive drugs in Chinese patients and determine the compliance level of treatment guidelines.

**Materials and methods:**

Around 11.1 million patients who received a prescription for antihypertensive therapy between January 2021 to December 2021 were obtained from a database of Hangzhou Kang Sheng Health Consulting CO., Ltd.

**Results:**

The mean age of hypertensive patients was 54.75 ± 12.98 years. About 6.7 million (60.30%) were males. About 46.07% of patients had comorbidities. The most common classes of antihypertensive medications used were calcium channel blockers (CCBs) and angiotensin II receptor blockers (ARBs). Around 78.33% of participants were on monotherapy. Diuretics + ARBs and Diuretics + CCBs + ARBs were the most commonly prescribed pattern in two-drug combination therapy and three-drug combination therapy, respectively.

**Conclusions:**

CCBs and ARBs were the two most frequently prescribed for patients with hypertension. The prescription pattern of antihypertensive medications in the study largely complied with recommended Chinese hypertension guidelines.

**Key messages**
- Cardiovascular disease is the most common complication of hypertension.
- Calcium channel blockers (CCBs) and angiotensin II receptor blockers (ARBs) are the two most commonly used drugs for hypertension patients in China.
- The proportion of combination prescription pattern in Chinese hypertensive patients is low.

## Introduction

Heart disease, stroke and kidney failure are all linked to hypertension, the most frequent risk factor [1]. It has been reported that nearly 9.4 million deaths happened over the world every year due to hypertension [2]. Hypertension has been a worldwide public health challenge. More than one billion adults are estimated to have high blood pressure, while the current worldwide hypertension control rate is only 31.7% [3]. It is predicted that by 2025, the number of people living with high blood pressure will reach 2 billion globally [4]. At the same time, China has a large number of patients with high blood pressure. A nationwide survey conducted in mainland China showed there was a significant increase in the prevalence of hypertension among adults aged 20 years and older from 25.7% in 2007 to 31.5% in 2017 [5]. Meanwhile, data from another national survey of Chinese adults aged 35 to 75 from 2014 to 2017 indicated the overall prevalence of hypertension was 44.7% [6]. The high prevalence of hypertension is placing an increasing burden on society and families. An earlier foreign study showed that high blood pressure and cardiovascular disease cost low- and middle-income countries 4% of their gross domestic product (GDP) a year, equivalent to $500 billion [7].

Some guidelines for the management of hypertension such as Joint National Committee (JNC)-8 guidelines [8] or ASH/ISH guidelines [9] have been developed worldwide and serve as reference standards for clinicians. Accordingly, the Chinese government and public health agencies have also issued a series of policy documents and guidelines on hypertension management such as 2018 Chinese guidelines [10] and 2020 revised national clinical practice guidelines for the management of hypertension [11], which recommended different antihypertensive drugs for patients with or without complications and contained the blood pressure level to be achieved. In general, the monotherapy was recommended first by guideline at the beginning of hypertension drug treatment and two-drug combination therapy was initiated when the single dose reached its maximum. Convert to another antihypertensive medication or use a three- or four-drug combination therapy to achieve better blood pressure control [10,11].

Clinical evidence has suggested that antihypertensive drugs can reduce the risk of heart failure, myocardial infarction, stroke and end-stage renal disease in hypertensive patients [12]. The latest current Chinese guidelines recommended angiotensin II receptor blockers (ARBs), calcium channel blockers (CCBs), beta-blockers (BBs), angiotensin-converting enzyme inhibitors (ACEIs) and thiazide diuretics; as first choice of drugs for patients with hypertension. This is following JNC guidelines [8]. However, many clinicians usually practice prescribing patterns to treat hypertensive patients based on their own clinical experiences rather than scientific evidence. In addition, to our knowledge, the research based on a real-world prescription pattern of antihypertensive drugs in patients diagnosed with hypertension in China is limited to date. Consequently, we conducted this retrospective study based on real-world data to describe the pattern of antihypertensive drugs prescribing among hypertensive patients in China and assess adherence with guidelines for hypertension. This study will provide forward guidance for the rational application of antihypertensive drugs in clinical practice and serve as a basis for future improving the baseline of hypertension management.

## Materials and methods

### Data source

This study was conducted retrospectively using data from Hangzhou Kangsheng Health Consulting Co., Ltd., which has so far provided chronic disease management services to more than 2000 hospitals and 150,000 pharmacies in Chinese cities. Patient’s name, age, gender, diagnosis and prescription were stored in the database (including prescription dates). The current study was a cross-sectional analysis of the prescribing practices of all patients with a diagnosis of hypertension who received at least one prescription for antihypertensive therapy between January 2021 to December 2021.

### Patients

**Figure 1.**
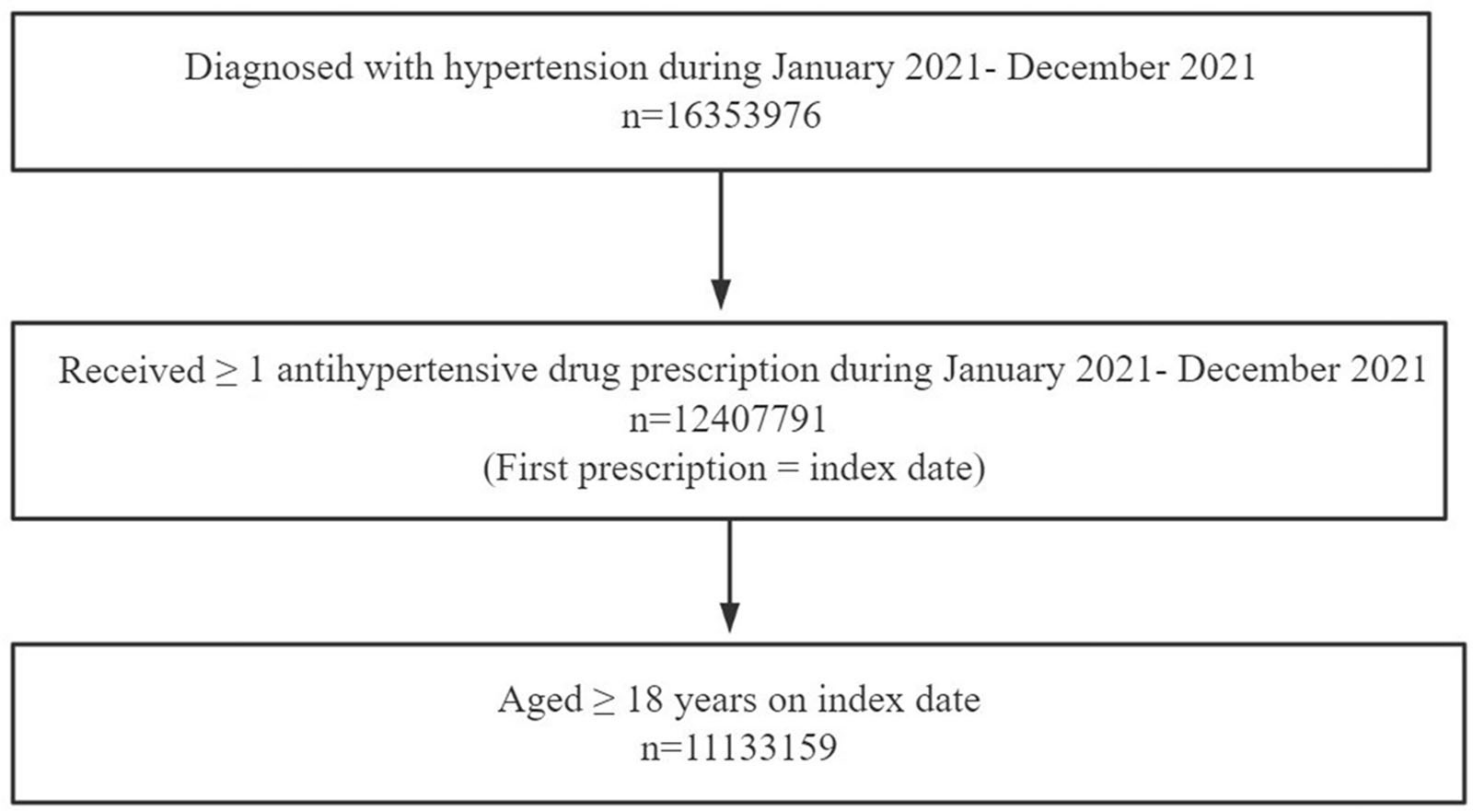
Patient disposition.

The study concentrated on hypertensive patients with a specific suggestion for the choice of antihypertensive drug use. All patients included in this study had previously received an outpatient or inpatient hypertension diagnosis from their physicians at the hospital in both public and private primary care settings. To meet the inclusion criteria, patients were required to have a diagnosis of hypertension and a prescription for antihypertensive therapy within the period from January 2021 to December 2021. Hypertensive patients with comorbidities were also included in our study and comorbidities were identified based on diagnostic information provided with prescriptions. Moreover, patients had to be 18 years of age or older on the date of the index prescription to participate in the study (Figure 1).

### Ethical consideration

The study used the database from Hangzhou Kang Sheng Health Consulting CO., Ltd, China. To guarantee patients’ privacy and confidentiality, all the data including protected information was encrypted before being analysed, ensuring that no health information could be linked to a specific person. As stated in the Ethical Guidelines for Epidemiological Research issued by the Office of Medical Ethics Expert Committee of the China National Health Commission, ethics approval and informed permission were not required for this study.

### Statistics

We used Structured Query Language to get data from a database. The variables analysed were diagnosis of patients, gender, age of hypertensive patients, classes of drugs and type of therapy (monotherapy or polytherapy). The first prescription of hypertensive patients between January 2021 and December 2021 was the index prescription and it was this prescription that was used in the analysis. The prescription proportion during the index period (January 2021 to December 2021) was the endpoint.

The mean ± standard deviation was used for numerical data such as patients’ age or total number of drugs prescribed for patients of hypertension. The numbers and percentages were used for categorical data such as gender or prescribing pattern. Prescription of five class of antihypertensive drugs were reported as percentages in the study population. Python 3.7 was used for all statistical analyses.

## Results

### Clinical characteristics of hypertension patients

The clinical characteristics of hypertensive patients were shown in Table 1. At last, a total of 11,133,159 patients met the inclusion criteria for the cross-sectional analysis with the average age as 54.75 ± 12.98. Of the around 11.1 million participants, about 4.4 million (39.70%) were females. There were 53.93% of patients diagnosed hypertension only with no comorbidities. The top five most common diagnoses in the overall population were exhibited in Table 1. The most frequent complication was coronary heart disease (1.2 million, 10.89%), while about 332,287 (3.00%) hypertensive patients had diabetes mellitus, 226,318 (2.03%) patients had concomitant hyperlipidaemia and 144,391 (1.30%) patients accompanied by coronary heart disease and hyperlipidaemia. Concerning antihypertensive drugs, the mean number of antihypertensive drugs prescribed in the index prescription was 1.88 ± 1.11 during January 2021 to December 2021 (Table 1).

**Table 1.** Clinical characteristics of hypertension patient.

Characteristic	Overall population *N* = 11,133,159
Age(years)	54.75 ± 12.98
Male gender (n, %)	6,712,910 (60.30)
Diagnosis (Top 5)	
Hypertension (n, %)	6,004,296 (53.93)
Hypertension + coronary heart disease (n, %)	1,211,916 (10.89)
Hypertension + diabetes mellitus (n, %)	334,287 (3.00)
Hypertension + hyperlipidaemia (n, %)	226,318 (2.03)
Hypertension + coronary heart disease + hyperlipidaemia (n, %)	144,391 (1.30)
Number of antihypertensive drugs in index prescription	1.88 ± 1.11

The gender and age distribution of hypertensive patients were shown in Table 2. For the overall population, the youngest age was 18 years old and the oldest was 100 years old. Among them, a large majority of participants were between 40 and 65 years old, accounting for 63.04% of the patients.

**Table 2.** Distribution of patient’s gender and age.

Age (years)	<40	40 ∼ 65	>65
Male (n, %)	720,759 (50.11)	4,290,486 (61.13)	1,701,665 (63.59)
Female (n, %)	717,612 (49.89)	2,728,408 (38.87)	974,299 (36.41)
All (n, %)	1,438,371 (12.92)	7,018,894 (63.04)	2,675,894 (24.04)

### Index prescription proportions

**Figure 2.**
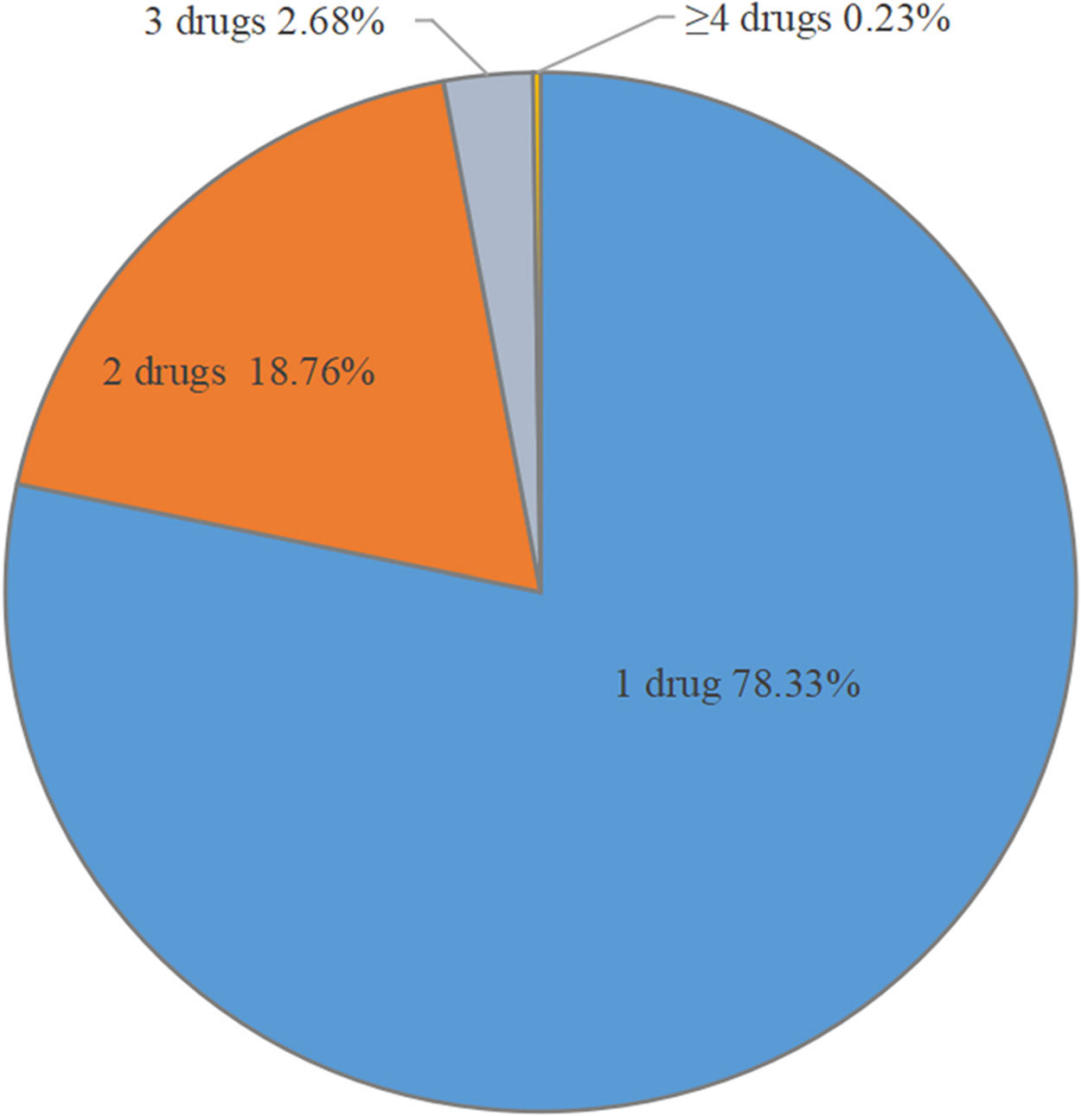
Single drug therapy and 2-, 3- and ≥4-drug combinations in the overall population.

Of the 11.1 million patients, 78.33% of the patients were on monotherapy, 18.76% were on dual therapy, 2.68% were on triple therapy and 0.23% were on four or more antihypertensive drugs therapy (Figure 2).

Table 3 shows the medication regimen for patients with hypertension of different genders and ages in detail. Among the 11.1 million prescriptions, monotherapy was the most common therapy among different age groups and gender. The prescription proportions of combined use of more than 2 drugs in the male and the female population was 22.21% and 20.85%, respectively.

**Table 3.** Combination of antihypertensive drugs in patients of different gender and age.

Variable	Monotherapy (n, %)	Drug combinations (n, %)	Drug combinations (n, %)	≥4 Drug combinations (n, %)
Gender				
Male	5,222,212 (77.79)	1,288,269 (19.19)	186,498 (2.78)	15,931 (0.24)
Female	3,498,405 (79.15)	800,926 (18.12)	111,741 (2.53)	9177 (0.21)
Age (years)				
<40	1,130,001 (78.56)	266,067 (18.50)	38,903 (2.70)	3400 (0.24)
40 ∼ 65	5,496,673 (78.31)	1,319,500 (18.80)	187,032 (2.66)	15,689 (0.22)
>65	2,093,943 (78.25)	503,628 (18.82)	72,304 (2.70)	6019 (0.22)

**Figure 3.**
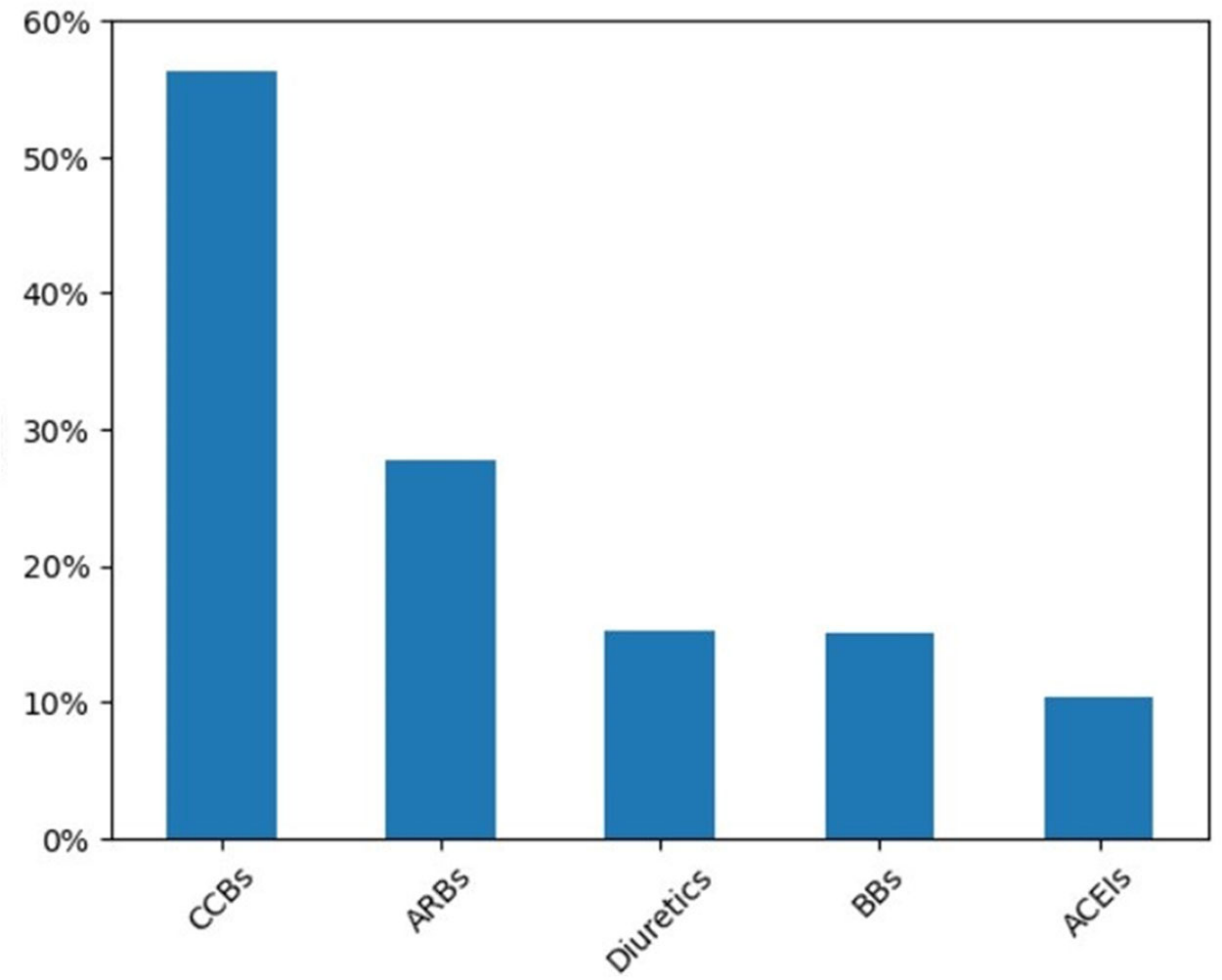
Proportion of each class of antihypertensive drug used in the overall population.

Figure 3 displays the prescription proportions of five classes of antihypertensive drugs guided by clinical guidelines of China as indicator prescriptions in the overall patients receiving single or combined antihypertensive therapy. As the results showed that CCBs were the most frequently used antihypertensive drug in the patients (56.35%), followed by ARBs (27.76%), thiazide diuretics (15.32%), BBs (15.03%) and ACEIs (10.34%).

### Monotherapy

**Figure 4.**
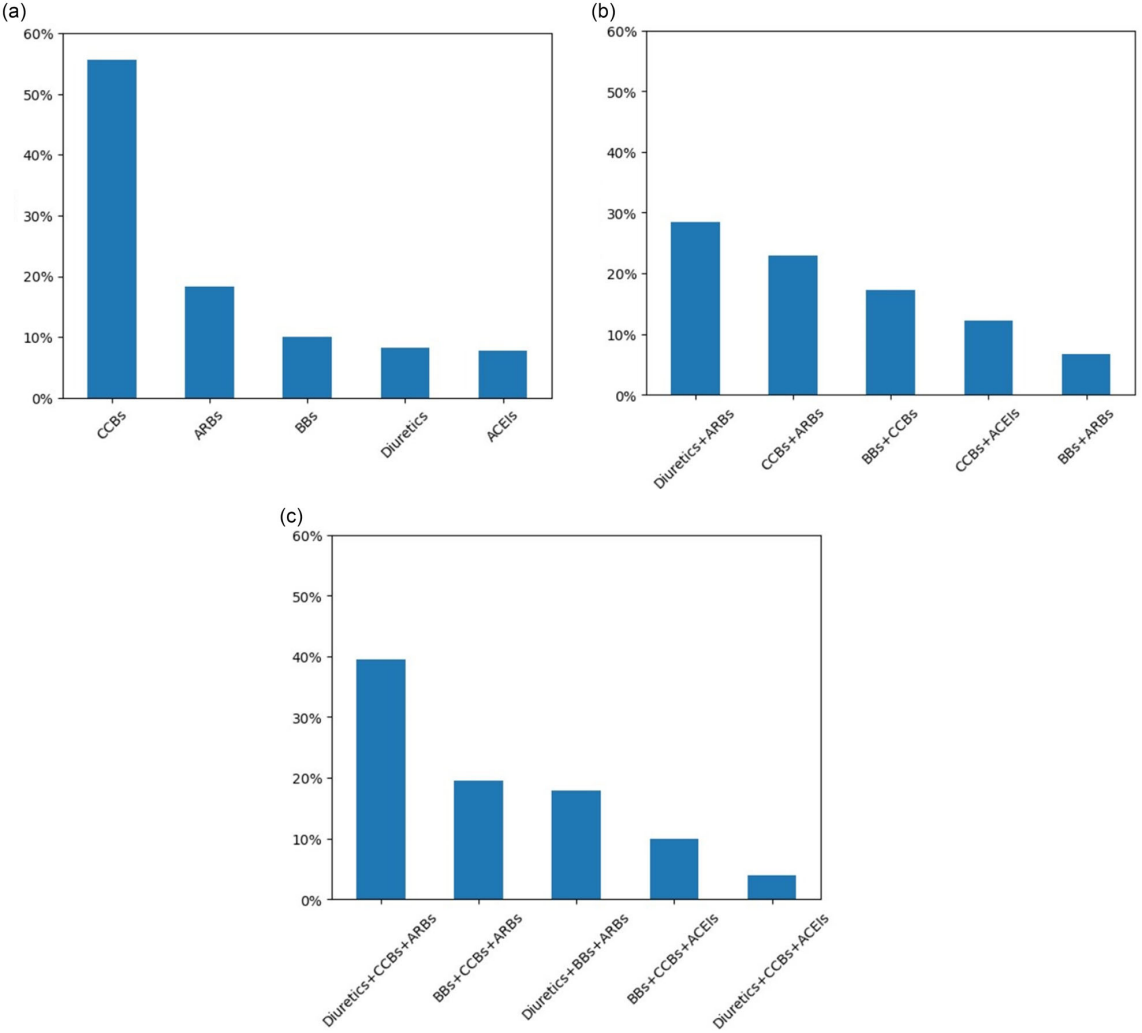
(a) Proportion of each drug used as antihypertensive monotherapy. (b) Proportion of each scheme used for antihypertensive treatment in combination of two drugs (five most common combinations). (c) Proportion of each scheme used for antihypertensive treatment in three-drug combination (five most common combinations).

Prescription proportions of antihypertensive drugs prescribed for the included patients who received monotherapy were shown in Figure 4(a). There were about 8.7 million patients around 78% of the overall population who accepted one drug therapy. The most frequently prescribed antihypertensive drug in monotherapy was CCBs (55.64%), followed by ARBs (18.30%), BBs (10.05%), thiazide diuretics (8.24%) and ACEIs (7.78%).

### 2-Drug combination therapy

The top five 2-drug treatment combinations with the highest prescribing proportions in the general population were shown in Figure 4(b). There were about 2.1 million patients around 19% of the population who received a two-drug combination. Diuretics + ARBs (28.42%) were the most frequently prescribed for patients with dual prescriptions, while the use of BBs + ARBs (6.63%) was lower than that of the other antihypertensive drugs.

### 3-Drug combination therapy

The top five 3-drug treatment combinations with the highest prescribing proportions in the general population were shown in Figure 4(c). There were 298,239 patients around 3% of the population who received a three-drug combination. The most commonly prescribed combination across all the patients was Diuretics + CCBs + ARBs which accounted for 39.45% of the three-drug therapy. This was followed by BBs + CCBs + ARBs in 19.50% of patients.

## Discussion

This study based on a real database displayed the actual situation of antihypertensive prescription patterns in Chinese hypertensive patients. The analysis focussed on five categories of antihypertensive drugs suggested by Chinese guidelines: calcium channel blockers (CCBs), angiotensin-converting enzyme inhibitors (ACEIs), angiotensin II receptor blockers (ARBs), beta-blockers (BBs) and thiazide diuretics. The present study mirroring a previous study by Cui et al. [13] displayed that coronary heart disease was the most common comorbidity. It was not surprising since hypertension and coronary heart disease often coexisted due to a similar range of risk factors.

In recent years, the Chinese government has made significant efforts to management of hypertension, including fostering the construction of basic medical centres and educated general medical practitioners, as well as adopting a basic health insurance programme for all inhabitants [14], so that the treatment and control rates have shown an upward trend. However, the control rates of hypertension patients in China is still much lower than that in developed countries [15]. For example, the data of the National Health and Nutrition Examination Survey in United States showed that the overall control rate of hypertension was 31.8% in 1999-2000, 48.5% in 2007-2008, 53.8% in 2013-2014 and 43.7% in 2017-2018, respectively [16]. Alternatively, a national survey in China displayed the control rate of hypertension among Chinese adults was 6.1% in 2002 and 11.2% in 2010 [17]. Apart from that, another large epidemiological survey containing 1.7 million community-dwelling adults from all 31 provinces in mainland China reported that 30.1% of the study population received prescribed antihypertensive medications, but the people who had their blood pressure under control only accounted for 7.2% [6].

Many previous studies [18,19] conducted in China have shown that the prevalence of hypertension was higher among males compared to females. Our study indicated that males were more likely to receive combination therapy compared to females. There were several reasons for this result. Firstly, this may be because males preferred to have unhealthy lifestyles such as smoking or drinking leading to higher rates of complications in their later life than females, so they needed more drugs to achieve optimal blood pressure [20–22]. Secondly, females were usually more patient, more prone to be aware of their hypertensive status and had better adherence to antihypertensive drugs than males [23,24].

In a review on prescribing patterns of antihypertensive drugs, Jarari et al. [25] reported that hypertensive patients in India were generally treated with at least two drugs. This multiple therapy was recommended by the guidelines, which suggested that small doses of different classes of antihypertensive drugs were more helpful than a single large dose. In addition, a previous study [26] has shown that monotherapy was often insufficient in hypertension management and most hypertensive patients required more than one substance class especially in the presence of comorbidity to reach their goal blood pressure. However, our study observed that majority of subjects accounting for around 78% of general patients currently used monotherapy and the mean number of antihypertensive drugs prescribed in the present study was 1.88 ± 1.11. The prescription rate of one drug therapy was comparable to the finding of an earlier nationwide hypertension survey which reported about 70% of treated hypertensive patients used one drug for individuals with hypertension [27] but significantly higher than that of foreign countries with the prescription rate of monotherapy as 53.1% in Malaysia [28] and 55.4% in Germany [29]. According to JNC 8 guideline [8], patients with hypertension were recommended to receive combination therapy from the first prescription. Our finding demonstrated that combination therapy was scarce in patients with hypertension in China which was in agreement with another research [6]. Considering the relatively low proportion of patients reaching target blood pressure level control and the high efficacy of combined antihypertensive therapy, significant efforts to increase the proportion of initial combination therapy may be urgently needed.

In a retrospective observational study of older hypertensive patients conducted by Ohishi et al. [30] in Japan, CCBs and ARBs were reported to be the most commonly used drugs in the hypertensive population. In line with the finding of their research, our study also found that CCBs (56.35%) and ARBs (27.76%) were the most frequently used drugs regardless of the initial treatment. Beyond that, in a similar patient population, Xu et al. [31] observed that the consumption of the five classes of antihypertensive drugs in China nearly doubled from 2007 to 2012 and CCBs and ARBs were the most frequently prescribed antihypertensive drug classes. According to Chinese guidelines [10,11], this may be due to that CCBs have a strong antihypertensive effect, good tolerance, no absolute contraindication, relatively wide scope of application and is more suitable for simple systolic hypertension in the elderly. Additionally, the relatively low ACEI prescriptions may reflect side effects such as dry cough, which are common in Asian populations [32]. The decrease in the use of ACEIs may be responsible for the increase in the use of alternative drug ARBs.

Conversely, BBs were still frequently prescribed together with CCBs, ARBs or diuretics in this study. This finding conflicted with some guidelines, which did not suggest BBs as a first-line antihypertensive drug [33]. In a meta-analysis, Larochelle et al. [34] found that among patients with hypertension Beta-blockers could significantly lower the incidence and death from cardiovascular events in young individuals but increased the risk of stroke in the elderly. Meanwhile, the European Hypertension Guidelines do not recommend beta-blockers for stroke prevention [35], but they should be used first if there is a specific indication such as coronary heart disease, especially after myocardial infarction, congestive heart failure or tachyarrhythmia [36]. Therefore, the fact in this study may be explained by the larger proportion of individuals with heart related diseases.

Although thiazide-type diuretics were recommended as initial therapy for most patients [37], it was observed that the prescription proportion was fairly lower than that of ARBs and CCBs in the current study. The low preference for diuretics may be related to their adverse effects such as hypokalaemia. Diuretics have been recognized as an essential medicine for intensive hypertensive treatment. In a review, Sato et al. [38] have demonstrated that ARBs-based combination therapies with either CCBs or diuretics were well tolerated and effectively lower the BP throughout a 24-hour interval by their long-acting half-lives, nighttime BP lowering effect and improving adherence. A similar result was reported by a randomized controlled experiment in Japan [39]. What’s more, a sub analysis of a clinical study has also revealed that combining ARBs with diuretics may produce better cardiovascular outcomes than combining an ARB with BBs, even in individuals with poor blood pressure control [40]. Also, the present study showed that Diuretics + ARBs and Diuretics + CCBs + ARBs were the most commonly prescribed pattern in two-drug combination therapy and three-drug combination therapy respectively, which reconfirmed these results.

There were several limitations to be considered in this study. Firstly, this was a single-centre study in the sense that the data came from a single database. As a result, this study may not be representative of all Chinese hypertensive patients. Future research should involve multi-centre investigation to strengthen the generalizability of the findings. Secondly, although we examined prescription patterns for hypertensive patients, the antihypertensive drugs prescribed may be appropriate for other conditions rather than hypertension [41–43]. Finally, because the value of blood pressure was not entered in the database, our study did not take into account the dosages of antihypertensive medications taken by patients or the level of blood pressure control they achieved.

## Conclusions

In summary, this study thoroughly analysed the prescription status of antihypertensive drugs in Chinese hypertensive patients. Most of hypertensive patients were on monotherapy. CCBs and ARBs were the most frequently prescribed for patients with hypertension. Even though the prescription pattern of antihypertensive medications in the study largely complied with recommended guidelines, there is still a need to strengthen health systems for effective hypertension management and patient education to ensure active participation in long-term care. Consequently, this real-world data analysis may be an indicator of the extent to which guidelines have infiltrated actual clinical practice. Moreover, the results of this study will provide insight into current management practices in both public and private primary care settings for older patients with hypertension. Detailed analyses including patients’ doses of medication as well as diseases history of patients can provide valuable information for considering the selection of appropriate treatment for each patient. Additional research, particularly focussing on the comorbidities of hypertension patients, is also needed in the future.

## Data Availability

The datasets used and/or analysed during the current study are not publicly available, but are available from the corresponding author on reasonable request.
